# What are risk factors for COVID-19 vaccine breakthrough infections in patients with previous history of bariatric surgery?

**DOI:** 10.17179/excli2022-4873

**Published:** 2022-05-12

**Authors:** Gerardo Sarno, Pietro Calabrese, Luigi Schiavo, Francesco Izzo, Vincenzo Pilone

**Affiliations:** 1 "San Giovanni di Dio e Ruggi D'Aragona" University Hospital, Scuola Medica Salernitana - Salerno, Italy; 2 Center of Excellence of Bariatric Surgery of the Italian Society of Obesity Surgery and Metabolic Disease (SICOB), Unit of General and Emergency Surgery, University Hospital San Giovanni di Dio e Ruggi d'Aragona, P.O. Gaetano FucitoMercato San Severino, Salerno, Italy; 3 Department of Medicine, Surgery and Dentistry, “Scuola Medica Salernitana”, University of Salerno, Baronissi, Salerno, Italy; 4 Istituto Nazionale per lo Studio e la Cura dei Tumori di Napoli, Fondazione "G. Pascale", 80131 Naples, Italy

## ⁯

COVID-19 is still a worldwide pandemic burden. Until now there have been globally 434.154.739 confirmed cases of COVID-19, including 5.944.342 deaths, reported to WHO (WHO, 2022[10]). Several attempts to find a specific pharmacological therapy for the treatment of COVID-19 have been carried out since the begin of the pandemia. Antivirals have shown more effectiveness when administered early in the disease course, but no antiviral treatment demonstrated efficacy at reducing COVID-19 mortality.

Vaccination is the key strategy to stop the COVID-19 pandemic. Several vaccines are nowadays available, each of them appears to be safe and effective in preventing severe COVID-19, hospitalization, and death against all variants of concern. Also the benefits of COVID-19 vaccination outweigh the risks, despite rare, of serious adverse effects (Fiolet et al., 2012[4]). As a matter of fact until now out of a total of 10.585.766.316 vaccine doses have been administered (WHO, 2022[10])*.* Italy has been the first European country dealing with the spread of COVID-19 outside China, and as for 01^st^ March 2022 the total number of accessed cases in Italy was 12.782.836 with 154.767 deaths (although not yet confirmed upon certification of cause of death by the “Istituto Superiore di Sanità” ), (Italian National Institute of Health, 2022[6])*, *and at the same time the vaccination cycle was completed in 89.32 % of the population aged 12 years and more, with a total rate of people vaccinated or with a recovery from the disease of 94.1 % (Italian National Institute of Health, 2022[5]). Emerging reports described breakthrough SARS-CoV-2 infections in fully vaccinated individuals. The main causes of vaccine-breakthrough COVID-19 infections have been found in pre-existing comorbidities and elderly. Among comorbidities overweight, type 2 diabetes mellitus, blood hypertension, cardiovascular and chronic lung diseases were associated to an increased risk of severe/critical disease especially in patients with four of more comorbidities (Butt et al., 2021[2])*. *In this view patients with history of bariatric surgery may be potentially at major risk of breakthrough because they were previously affected by severe obesity that usually predisposes to multiple comorbidities, especially metabolic derangements and cardiovascular impairment, as well as an increased susceptibility to infections.

Thus, we investigated: 1) the prevalence of COVID-19 vaccine breakthrough; 2) the effect of the timing and the surgical procedure employed on the risk for COVID-19 vaccine breakthrough and infection; 3) the clinical manifestations of COVID-19 vaccine breakthrough in bariatric patients.

This study was carried out from February 01^st^, 2022 to February 28^th^ 2022. All research methods were performed in accordance with the relevant guidelines and regulations of the Declaration of Helsinki. The target participants were adults aged 18 years and above undergone bariatric intervention due to morbid obesity. Due to the limitations in employing face-to-face methods during an active out-break, the data were collected using the Google Form platform via an online questionnaire. A snowball sampling strategy was used to distribute the online questionnaire via social media (Whatsapp, Facebook). The participants were then asked to share the questionnaire link to individuals in their social circles. These social media platforms were chosen because of their wide use worldwide. 

The questionnaire was developed through a literature review. The survey form consists of 16 questions covering socio-demographic and anthropometric characteristics (Questions 1 to 6) , intervention details (Questions 7 to 10), outcome (question 11), SARS-CoV-2 vaccination status (Question 12), evidence and timing of infection (Questions 13 to 15), clinical characteristics of patients with previous history of bariatric interventions developing vaccine breakthrough COVID-19 infection (Question 16). Question 16 was only answered by respondents who responded 'yes' to Question 13 “*Did you test positive for COVID-19?*”.

Socio-demographic and anthropometric characteristics include: age in years, gender, weight (kg), height (cm), BMI (kg/m^2^). Intervention details were consistent in type, date and technique (open or laparoscopic) employed. Weight loss was considered as stable weight achieved over the follow-up. Information regarding SARS-CoV-2 vaccination status were the specific vaccine type and the numbers of administered doses. Finally, questions 13-16 addressed the clinical aspects of COVID-19 disease such as the timing of test positive for COVID-19 and clinical aspects (no symptoms, respiratory failure, myalgias, cold, fever, sore throat, headache, loss of smell-taste perception, asthenia, gastrointestinal symptoms, hospitalization). The answers to the questions 7, 9 and 13 were in dichotomous 'yes' or 'no' format. The original questionnaire was developed in English and later translated to Italian using forward and back translation. A pilot assessment was conducted among the general population through snowball sampling in order to ensure the questions were clearly written, easily understood and unambiguous. In the aim to highlight the features of patients with vaccine breakthrough and COVID-19 infection, patients who completed the vaccination course, were compared with those without evidence of COVID-19. 

Results have been described as mean ± SD or percentage/number. The differences of means ± SD of age, weight, height, and BMI according to COVID-19 yes or no were analyzed by Student's unpaired t-test. The chi square (χ^2^) test was used to evaluate the differences in the frequency distribution of categorical variables included in this study. Data were analyzed using the SPSS Software (PASW Version 21.0, SPSS Inc., Chicago, IL, USA) and MedCalc® package (Version 12.3.0 1993-2012 MedCalc Software bvba-MedCalc Software, Mariakerke, Belgium).

A total of 170 respondents answered the questionnaire. Among them 6 (3.5 %) were undergone non interventional bariatric procedures (e.g. intragastric ballon placement), 5 (2.9 %) reported COVID-19 infection before surgery, 21 (12.4 %) were not vaccinated and so excluded.

Thus the study cohort consisted of 138 patients, 6 (4.3 %) males, 132 (95.7 %) females (mean age 46.95 ± 9.48 years; weight 81.18 ± 21.15 kg; height 163.72 ± 7.03 cm; BMI: 30.20 ± 7.05 kg/m^2^). The majority of surgical interventions was performed through a laparoscopic approach: 120 (86.95 %) vs 18 (13.05 %) open procedures. Malabsorptive interventions were detected in 86 (62.3 %) patients while restrictive procedures in 52 (37.7 %) patients. The mean weight loss after surgery was 48.79 ± 20.53 kg. COVID-19 infection was reported in 48 (34.8 %) patients. Operative details, timing of intervention, outcome and incidence of COVID-19 infections are described in the supplementary information (Table 1). All of the non-COVID patients reported a complete vaccination course. Of the 48 patients in whom COVID-19 infection was detected, 3 (6.25 %) received one dose of vaccine, while 45 (93.75 %) completed the vaccination course (19 received two vaccine doses, 26 also received a third dose “dose booster”). By considering only fully vaccinated patients, symptoms were reported by 31 (68.9 %). Although one or more symptoms occurred simultaneously: headache, flu and asthenia were the most frequently reported. Among symptomatic, patients requiring hospitalization were 7 (22.6 %), all of them recovered from symptoms without any further complications (vaccines status and COVID-19 related symptoms are described in the supplementary information, Table 2).

When compared to non COVID-19, in patients with COVID-19 vaccine breakthrough and complete vaccination, the prevalence of malabsorptive surgery was significantly higher (p= 0.04) as well as an open surgical approach (p=0.03). Also a significantly higher post-operative weight loss was recorded in patients suffering from vaccine breakthrough (p= 0.03), while no differences were noted according to gender, age BMI, and the timing of intervention (supplementary information, Table 3). Finally, we investigated if the kind of intervention performed could have had an impact on clinical manifestations of COVID--19. Among symptomatic COVID-19 vaccine breakthrough patients, a comparison between patients undergone malabsorptive and patients undergone restrictive interventions did not evidence differences in the prevalence of both clinical manifestations and need of hospitalizations (supplementary information, Table 4). 

COVID-19 vaccine breakthrough is a matter of concern and data regarding this specific condition are still lacking. Vaccines have been reported to be effective in reducing risk of getting COVID-19 infections by 70-90 % and shield from severe infections. It has been reported that subjects with obesity are more likely to develop COVID-19 vaccine breakthrough, but at the best of our knowledge this letter is the first describing this evidence in a cohort of patients with history of severe obesity already undergone bariatric surgery. In this report the prevalence of COVID-19 vaccine breakthrough was 34.8 % in patients undergone at least one vaccine dose. By excluding these last patients and considering only those with a complete vaccination course the incidence accounted for 33.4 %. Our findings are of particular interest since up to one third may experience a COVID-19 infection in spite of a complete vaccination course. Moreover, we found an increase in COVID-19 susceptibility in patients undergone malabsorptive interventions, and open interventions, irrespectively of timing of surgery. The higher prevalence of COVID-19 vaccine breakthrough in our cohort is mostly due to the fact that most of them were still affected by obesity after surgery. As well-known obesity is an Achille's heel for infections because of several reasons.

First, subjects with obesity are often affected by immune dysfunction that results in a poor seroconversion upon some vaccine administration, together with increased risk of infection even when the seroconversion seems robust (Neidich et al., 2017[8]). Indeed, a recent clinical trial has shown that higher BMI is associated with lower Ab titers in response to COVID‐19 vaccine in 248 Italian healthcare workers (Pellini et al., 2017[9]). Interestingly, we found that BMI per se is not associated with a higher risk to get SARS-CoV-2 infection but the “history” of weight loss does. 

Indeed, it is well-known that all bariatric procedures, although with variable degrees alter both anatomy and physiology of the gastrointestinal tract. However, nutritional deficiencies are more frequently detected after malabsorptive interventions and due to reduced food intake and malabsorption. In patients undergoing to malabsorptive interventions the most common nutritional deficits are represented by vitamin D and iron deficiency. Vitamin D has been acknowledged as an immunomodulatory hormone with various degree of activity, among them a protective role against various upper respiratory tract infections. Also vitamin D can inhibit hyperinflammatory reactions and facilitate healing process in the lung tissue. Vitamin D enhances innate cellular immunity by the induction of antimicrobial peptides, like cathelicidin and defensins and hypovitaminosis D was found to correlate with severity of respiratory failure and mortality in patients affected by COVID-19 (Daneshkhah et al., 2020[3]). On the other hand the diagnosis of iron deficiency ranges between 33 % and 49 % among patients undergone bariatric procedures within two years after surgery, as reported by the American Society of Hematology, and is more often detected in patient undergone malabsorptive interventions (American Society of Hematology, 2022[1]). Several factors influence the iron malabsorption such as hypocloridria, the by-passing of duodenum and proximal jejunum where iron is usually absorbed as well as reduced food intake and changes in food preferences with lower assumption of meat (Lupoli et al., 2017[7]). Anemia and iron deficiency are usually detected in patients with COVID-19, and have been associated with progression to severe COVID-19 course and even death. The evidence of an increase in prevalence of COVID-19 in patients undergone open approach may be explained with a more demolitive procedure or with somewhat surgical complication that could modify the gastrointestinal anatomy to the point that gets worse the *malabsorptive procedure-related* micronutrients deficiencies but the lack of operative details does not allow to draw a definitive picture. Conversely the advantage of the laparoscopic technique is widely acknowledged, and nowadays the vast majority of bariatric interventions are carried out through this approach in center of excellence with low rate of complications.

We are aware that there are some limitations in this report. First, data reported did not allow for any statements on a causal relationship regarding the SARS-CoV-2 vaccination status and bariatric surgery. 

In addition, although the sample size was small, this study provides insights on a new topic that could be used as background for furthers studies. In conclusion this report is the first to investigate risk factors predisposing patients undergone bariatric surgery to COVID-19 vaccine breakthrough. When malabsorptive interventions are performed a higher risk of vaccine breakthrough development has been recorded and particular attention should be paid to an accurate replacement of micronutrients in these very same patients. 

## Conflict of interest

The authors declare no conflict of interest.

## Supplementary Material

Supplementary information
