# Supplementary material for: What are risk factors for COVID-19 vaccine breakthrough infections in patients with previous history of bariatric surgery?
Source: EXCLI J. 2022 May 12;21:786–90. doi: 10.17179/excli2022-4873 (PMC9360469; doi:10.17179/excli2022-4873)
Supplement: Supplementary information [file EXCLI-21-786-s-001.pdf]

EXCLI Journal 2022;21:786-790 – ISSN 1611-2156
Received: March 24, 2022, accepted: May 11, 2022, published: May 12, 2022

Supplementary information to:

Letter to the editor:

WHAT ARE RISK FACTORS FOR COVID-19 VACCINE BREAKTHROUGH INFECTIONS IN PATIENTS WITH PREVIOUS HISTORY OF BARIATRIC SURGERY?

Gerardo Sarno MD, PhD, FACS*[1], Pietro Calabrese, MD[2], Luigi Schiavo, PhD, FACN[2,3], Francesco Izzo, MD[4], Vincenzo Pilone MD, PhD[2,3]

[1] “San Giovanni di Dio e Ruggi D’Aragona” University Hospital, Scuola Medica Salernitana - Salerno, Italy
[2] Center of Excellence of Bariatric Surgery of the Italian Society of Obesity Surgery and Metabolic Disease (SICOB), Unit of General and Emergency Surgery, University Hospital San Giovanni di Dio e Ruggi d’Aragona, P.O. Gaetano FucitoMercato San Severino, Salerno, Italy
[3] Department of Medicine, Surgery and Dentistry, “Scuola Medica Salernitana”, University of Salerno, Baronissi, Salerno, Italy
[4] Istituto Nazionale per lo Studio e la Cura dei Tumori di Napoli, Fondazione "G. Pascale", 80131 Naples, Italy

* **Corresponding author:** Gerardo Sarno, MD, PhD, FACS, “San Giovanni di Dio e Ruggi D’Aragona” University Hospital, Scuola Medica Salernitana, Via San Leonardo, 84125 - Salerno, Italy, Phone: +39.3394536396; E-mail: gerardo.sarno@sangiovannieruggi.it

https://dx.doi.org/10.17179/excli2022-4873

This is an Open Access article distributed under the terms of the Creative Commons Attribution License (http://creativecommons.org/licenses/by/4.0/).

EXCLI Journal 2022;21:786-790 – ISSN 1611-2156
Received: March 24, 2022, accepted: May 11, 2022, published: May 12, 2022

Table 1: Operative details, outcome and incidence of COVID-19 infections in **138 bariatric patients**

Intervention	Open<br>**18 (13.04 %)**	Laparoscopy<br>**120 (86.96 %)**
Gastric bending	0	3
Biliopancreatic diversion	10	7
Mini bypass	2	52
Roux-en-Y gastric bypass	4	11
Sleeve gastrectomy	2	47
Time of intervention		
< 1 year<br>1-5 years<br>> 5 years	25 (18.2 %)<br>64 (46.4 %)<br>49 (35.5 %)	
Weight loss	48.79 ± 20.53 kg	
COVID-19 infection		
Yes<br>No	48 (34.8 %)<br>90 (65.2 %)	

Results expressed as mean ± standard deviation

EXCLI Journal 2022;21:786-790 – ISSN 1611-2156
Received: March 24, 2022, accepted: May 11, 2022, published: May 12, 2022

Table 2: Vaccines status and COVID-19 related symptoms in 48 bariatric patients experiencing COVID-19 vaccine breakthrough.

Vaccination status	Patients
1 dose	3 (6.25 %)
2 doses	19 (39.6 %)
3 doses	26 (54.15 %)
**Symptoms in 45 patients with complete vaccination course**	
No	14 (31.1 %)
Yes*	31 (68.9 %)
Headache	26
Flu	24
Gastrointestinal disorders	9
Asthenia	27
Dyspnea	8
Fewer	16
Ageusia	12
Anosmia	10
**Hospitalization in 31 symptomatic COVID19 patients**	
No	24 (77.4 %)
Yes*	7 (22.6 %)

* One or more symptoms occurred simultaneously in the same patient

EXCLI Journal 2022;21:786-790 – ISSN 1611-2156
Received: March 24, 2022, accepted: May 11, 2022, published: May 12, 2022

Table 3: Comparison between 45 COVID-19 bariatric patients with complete vaccination status suffering vaccine breakthrough and 90 non COVID-19 bariatric patients

	**COVID breakthrough n = 45**	**Non-COVID n = 90**	***p***
**Sex**			
M	0	5	**X^2=0.78; p=0.37**
F	45	85	
**Age**	47.11± 9.80	47.5 ± 9.44	P=0.8235
**BMI**	28.4 ± 6.24	30.3 ± 7.08	P=0.12
**Type of surgery**			
Malabsorptive	33	50	**X^2=4.00; p=0.04**
Restrictive	12	40	
**Surgical approach**			
Open	10	8	**X^2=4.61; p=0.03**
Laparoscopy	35	82	
**Timing of intervention**			
< 1 year	6	19	**X^2=1.20; p=0.27**
1-5 years	22	41	**X^2=0.13; p=0.71**
> 5 years	21	30	**X^2=2.26; p=0.13**
**Weight loss**	51.9 ± 22.19	44.7 ± 16.03	P=0.03

Results expressed as mean ± standard deviation

Table 4: Comparison between 31 symptomatic COVID-19 bariatric patients with complete vaccination status according to the nature of the bariatric procedure.

	Malabsorptive **24**	Restrictive **7**	*p*
**Symptoms***			
Headache	20 (83.3 %)	6 (87.7 %)	X^2 =0.02; p=0.88
Flu	19 (79.2 %)	5 (71.4 %)	X^2 =0.18; p=0.66
Gastrointestinal disorders	7 (29.2 %)	2 (28.6 %)	X^2 =0.0009; p=0.97
Asthenia	21 (87.5 %)	6 (87.7 %)	X^2 =0.01; p=0.90
Dyspnea	8 (33.3 %)	0	X^2 =0.95; p=0.32
Fewer	13 (54.2 %)	3 (42.8 %)	X^2 =0.27; p=0.59
Ageusia	12 (50 %)	0	X^2 =2.83; p=0.09
Anosmia	10 (41.7 %)	0	X^2 =2.26; p=0.13
**Hospitalization**	6 (25 %)	1 (14.3 %)	X^2 =0.35; p=0.55

* One or more symptoms occurred simultaneously in the same patient
